# Postoperative Pulmonary Complications in Patients Undergoing Elective Thoracotomy Versus Thoracoscopic Surgeries

**DOI:** 10.7759/cureus.45367

**Published:** 2023-09-16

**Authors:** Iskander S Al-Githmi, Abdulrahman Alotaibi, Alaa Habeebullah, Weam Bajunaid, Sondos Jar, Nadin A Alharbi, Haneen Aziz

**Affiliations:** 1 Cardiothoracic Surgery, King Abdulaziz University Faculty of Medicine, Jeddah, SAU; 2 Thoracic Surgery, King Abdulaziz University Faculty of Medicine, Jeddah, SAU; 3 Faculty of Medicine, King Abdulaziz University Hospital, Jeddah, SAU

**Keywords:** postoperative complications, opioids, postoperative pulmonary complication, video-assisted thoracoscopic surgery, thoracotomy

## Abstract

Background

Postoperative pulmonary complications correlate highly with thoracic surgery compared to other surgeries. Video-assisted thoracoscopic surgery (VATS) is a minimally invasive surgical approach that provides considerable advantages over major open thoracotomy.

Methodology

This is a retrospective cohort study. All patients aged 18 years and above of both genders were included in the study. Cases following up outside King Abdulaziz University Hospital (KAUH), Jeddah, Saudi Arabia, were excluded from our study. Complications were measured per the records on follow-up day 1, day 7, and day 30. Mortality was measured within 30 days after the surgery.

Results

A total of 151 patients were included in the study. Age ranged from 18 to 85 years, with males representing 62.3% of the sample, while Saudis represented 59.6%. VATS was performed in 71.5%, while thoracotomy was performed in 28.5%. Of the total, 19.4% had postoperative complications within 30 days in the VATS group, while 23.3% were in the thoracotomy group. No significant differences were found between the rates of complications between the two groups. Additionally, the admission rate to ICU was significantly twice as common in the thoracotomy group (65.1%) compared to the VATS group (33.3%). Besides, the average duration of the chest tube's stay was three to seven days in both groups (62.1% in the VATS group and 70.7% in the thoracotomy group). Lastly, regarding the requirements of opioids, VATS showed more need for opioids (44.4%) compared to thoracotomy (32.6%).

Conclusion

The rates of postoperative complications were low in both groups, and no significant differences were found between the two procedures. In addition, the VATS group showed significantly higher use of opioids compared to the thoracotomy group. We recommend conducting further studies with larger sample sizes to increase the statistical power of detection.

## Introduction

Thoracic surgery is associated with a higher incidence of postoperative pulmonary complications (PPCs) due to its impact on respiratory function [1]. The European Joint Task Force guideline has identified specific PPCs in thoracic surgeries, including atelectasis, pneumothorax, acute respiratory distress syndrome, pneumonia, respiratory infection, pleural effusion, bronchospasm, pulmonary embolism, and respiratory failure [2].

Video-assisted thoracoscopy (VATS) is a minimally invasive surgical technique that utilizes a thoracoscope and a small video camera for various thoracic diseases [3]. Thoracotomy is a surgical technique that involves making incisions in the thorax cavity. This procedure is a major one and is associated with significant morbidity and mortality [4].

In contrast to thoracotomy, VATS is a minimally invasive technique that uses smaller incisions and causes minimal muscle damage without the need for rib retraction [5]. VATS has been increasingly used as an alternative to thoracotomy in various procedures including lobectomy and pneumothorax management due to its superior outcomes and fewer postoperative complications [6,7]. Previous studies have demonstrated that VATS is associated with better outcomes and fewer complications compared to thoracotomy.

In the United States, a cohort study was conducted using the Nationwide Inpatient Sample database to compare the outcomes of patients who underwent lobectomy using either thoracoscopic or open thoracotomy techniques. Thoracoscopic lobectomy was linked with fewer in-hospital postoperative sequelae, particularly supraventricular arrhythmias, myocardial infarction, pulmonary embolism, and empyema [5]. Additionally, VATS demonstrated better quality-of-life outcomes compared to thoracotomy [5].

In a 2017 study conducted in Spain, a cohort of cancer patients who underwent lobectomy surgery between January 2012 and January 2016 were examined [6]. Results showed that patients who underwent VATS experienced fewer PPCs and required less physiotherapy compared to those who underwent a traditional thoracotomy. Furthermore, a separate study was conducted comparing the duration of epidural analgesia and chest tube drainage between the two surgical procedures. Findings indicated that VATS resulted in a shorter period of both epidural analgesia and chest tube drainage in comparison to thoracotomy [8].

While several studies have found VATS to be superior to traditional thoracotomy for various surgical procedures, one study found no significant difference in surgical outcome or survival rates between the two methods for patients with suspected pulmonary metastasis [9]. Another retrospective study comparing VATS and thoracotomy in patients with oligometastatic pulmonary disease who underwent lung resection surgery found that both methods resulted in comparable pulmonary disease-free and overall survival rates [10].

Conversely, some studies have shown that thoracotomy is more effective than VATS for lymph node dissection in lung cancer patients. In a meta-analysis and systematic review of 29 articles that included both VATS and open lobectomy groups, open lobectomy was found to be more efficacious than VATS in treating lung cancer [10]. Additionally, thoracoscopy resulted in poorer overall survival rates in patients with a higher burden of oligometastatic pulmonary disease from osteosarcoma [10].

The literature comparing VATS and thoracotomy surgery is inconclusive due to inconsistent findings from previous studies. Moreover, there is a dearth of research conducted in Saudi Arabia on this topic. Therefore, the present study seeks to investigate and compare the incidence of short-term PPCs between VATS and thoracotomy at King Abdulaziz University Hospital (KAUH), Jeddah, Saudi Arabia. The primary objective is to provide empirical evidence to guide the selection of the optimal surgical procedure with respect to reducing the occurrence of pulmonary complications in the immediate postoperative period.

## Materials and methods

Study design

This is a retrospective cohort study that compares VATS and thoracotomy in terms of early postoperative complications and mortality. The duration of follow-up after surgery was determined to be within 30 days.

Study setting

This study was carried out in the Department of Thoracic Surgery at KAUH, which is considered one of the largest tertiary hospitals in the western region.

Study population

All patients who underwent VATS or thoracotomy, were aged 18 years and above, and of both genders were included in the study. Cases that continued follow-up outside KAUH were excluded from our study.

Data collection

The data were collected from the medical records of the patients. Records of surgeries from January 2018 to December 2022 were collected during a two-month period (January-February 2023). The investigators used Excel sheet for keeping and preparing the collected data for analysis.

Patients’ demographic information, anthropometric measurements, and comorbidities were collected from their baseline data in the records. Surgery variables included surgery type, conversion to thoracotomy, anesthesia, and duration of surgery. Postoperative variables included intensive care unit (ICU) admission, intubation, chest tube insertion, oxygen saturation, ejection fraction, and Mallampati score. Complications were measured per the records on follow-up day 1, day 7, and day 30. Mortality was measured within 30 days after the surgery.

Statistical analysis

The Statistical Package for Social Sciences (SPSS) Version 29.0 (IBM Corp., Armonk, NY) was used for the statistical analysis. Frequency tables and proportions were used for categorical variables summarization. The association between categorical variables was assessed using a chi-square test and Fisher exact test as appropriate according to the values of expected cells. Relative risk (RR) was calculated to compare between thoracotomy and VATS groups, considering thoracotomy as the exposure group of interest. The significance level was set at 0.05.

Ethical considerations

This research was approved by the Unit of Biomedical Ethics at the Faculty of Medicine at KAU (reference no 560-22). The data collection from the patient records was started after obtaining permission from the administration of KAUH. After the completion of data collection, medical record numbers were removed and replaced with serial numbers to keep patients' data secure. Anonymous data were sent for analysis and were used for research purposes only.

## Results

A total of 151 patients were included in the analysis. Age ranged from 18 to 85 years, with males representing 62.3% of the sample and females representing 37.7%, while Saudis represented 59.6%. Minor proportion of the participants had the following comorbidities: asthma (4%), chronic obstructive pulmonary disease (COPD) (2%), ischemic heart diseases (IHD) (2%), heart failure (HF) (2%), Duchenne muscular dystrophy (1.3%), interstitial lung diseases (1.3%), and cardiovascular accident (1.3%); pulmonary embolism, laryngeal carcinoma, pulmonary tuberculosis, bronchiectasis, patent ductus arteriosus, and HIV were present in 0.7% of the population each. VATS was performed in 71.5%, while thoracotomy was performed in 28.5%. The baseline characteristics of both groups are given in Table 1.

**Table 1:** Comparison of sociodemographic characteristics and diagnosis between VATS and thoracotomy patients BMI, body mass index; VATS, video-assisted thoracoscopic surgery

Variable	Groups	Surgical procedure				P-value
		VATS		Thoracotomy		
		N	%	N	%	
Age	≤55	81	75%	23	53.5	0.010
	>55	27	25%	20	46.5	
Gender	Male	68	63%	26	60.5%	0.772
	Female	40	37%	17	39.5%	
Nationality	Saudi	68	63%	22	51.2%	0.182
	Non-Saudi	40	37%	21	48.8%	
BMI	<18	19	29.7%	6	19.4%	0.284
	>25	45	70.3%	25	80.6%	
Smoking	Yes	29	26.9%	10	23.3%	0.786
	No	74	68.5%	30	69.8%	
	Not sure	5	4.6%	3	7%	
Diagnosis	Benign	74	68.5%	23	53.5%	0.327
	Malignant	34	31.5%	20	46.5%	

General anesthesia was used in all the patients in the two groups, while regional anesthesia was further administered in 20.5%. Of the total, 94.7% of the two groups needed chest tube insertion, 42.4% were admitted to ICU, and 13.9% were intubated after surgery. The surgery-related details are given in Table 2.

**Table 2:** Comparison of surgical and postoperative variables of the patients. ICU, intensive care unit; VATS, video-assisted thoracoscopic surgery **P-value calculated using the Fisher-Freeman-Halton exact test

Variables		Surgical procedure				P-value
		VATS		Thoracotomy		
		N	%	N	%	
Hospital stay	3 days or less	35	32.40%	3	7.00%	0.013
	4-6 days	25	23.10%	13	30.20%	
	7-10 days	22	20.40%	12	27.90%	
	More than 10 days	26	24.10%	15	34.90%	
ICU admission	Admitted	36	33.3%	28	65.1%	<0.001
	Not admitted	71	65.7%	13	30.2%	
	Missing	1	0.9%	2	4.7%	
Number of stay in ICU (days)	≤7 days	26	72.20%	19	67.90%	0.915**
	>7 days	8	22.20%	7	25.00%	
	Missing	2	5.60%	2	7.10%	
Endotracheal tube after surgery	Yes	7	6.50%	14	32.60%	<0.001>
	No	101	93.50%	29	67.44%	
Endotracheal tube duration	≤24 hours	1	14.3%	3	21.4%	0.687**
	>24 hours	6	85.7%	10	71.4%	
	Missing	0	0%	1	7.2%	
Chest tube insertion	Yes	103	95.37%	41	95.35%	1.000
	No	5	4.63%	2	4.65%	
Number of chest tube days	Less than 3 days	24	23.30%	1	2.33%	0.006
	3-7 days	64	62.1%	29	70.7%	
	Missing	15	14.6%	11	25.58%	

The Mallampati score showed class I in 18.5%, class II in 42.4%, class III in 7.9%, and class IV in 1.3%. During the surgery, oxygen saturation was above 95% in 86.8%, 95% in 3.3%, 93-94% in 5.3%, and less than 93% in 4.6%. The duration of surgery was less than 3 hours in 72.2%, 3 hours in 5.3%, and more than 3 hours in 22.5%.

Postoperative complications were assessed on days 1, 7, and 30; 3.3% of the two groups had postoperative complications at day 1, 17.9% had complications at day 7, and 2.6% had complications at day 30, while days postoperative complications within 30 days were present in 20.5%. The incidence of various complications at different time intervals after the surgeries was analyzed. The most common complications were emphysema and pleural effusion, with incidence rates of 0.70% on day 1 and 5.30% on day 7 for emphysema, and 2.60% on day 7 for pleural effusion. Other frequent complications included pneumothorax (0.70% on day 1 and 2.00% on day 7), and pain (1.30% on both day 1 and day 7). The detailed frequencies are given in Table 3. The presence of postoperative complications was compared between patients who underwent VATS and those who underwent thoracotomy; the results did not show statistical differences between the two modalities of surgery. The results are shown in Table 4.

**Table 3:** Postoperative complications in our patients.

Complication	Day 1	Day 7	Day 30
Pneumothorax	1 (0.70%)	3 (2.00%)	-
Emphysema	1 (0.70%)	8 (5.30%)	-
Pain	2 (1.30%)	2 (1.30%)	-
Pericarditis	1 (0.70%)	-	-
Pneumonia	-	3 (2.00%)	-
Empyema	-	2 (1.30%)	1 (0.70%)
Pleural effusion	-	4 (2.60%)	-
Wound infection	-	1 (0.70%)	-
Fever	-	3 (2.00%)	-
Hemothorax	-	2 (1.30%)	1 (0.70%)
Atrial fibrillation	-	1 (0.70%)	-
Hydropneumothorax	-	1 (0.70%)	-
Atelectasis	-	-	1 (0.70%)
Respiratory failure	-	-	1 (0.70%)
Septic shock	-	-	1 (0.70%)

**Table 4:** The risk ratios of postoperative complications among thoracotomy patients compared to VATS RR, relative risk; VATS, video-assisted thoracoscopic surgery Note: RR is calculated with the thoracotomy group as exposure

Duration	Yes/no	Procedure		RR
		VATS, n (%)	Thoracotomy, n (%)	
Postoperative complications on day 1	Yes	3 (2.8%)	2 (4.7%)	1.68
	No	105 (97.2%)	41 (95.3%)	0.623
Postoperative complications on day 7	Yes	17 (15.7%)	10 (23.3%)	1.48
	No	91 (84.3%)	33 (76.7%)	0.347
Postoperative complications on day 30	Yes	3 (2.8%)	1 (2.3%)	0.84
	No	105 (97.2%)	42 (97.7%)	1.000
Postoperative complications within 30 days	Yes	21 (19.4%)	10 (23.3%)	1.19
	No	87 (80.6%)	33 (76.7%)	0.657

**Figure 1:**
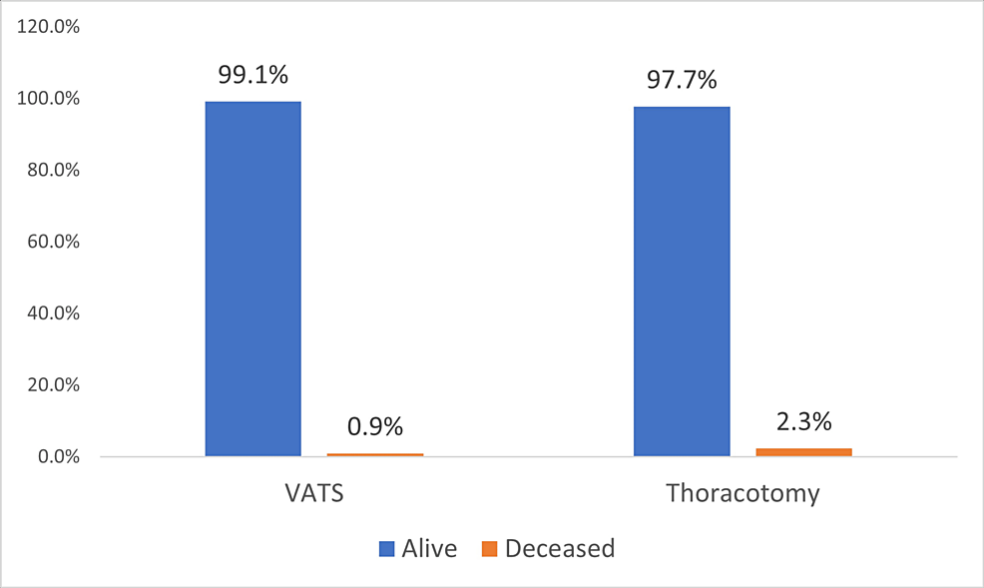
Postoperative mortality compared between VATS and thoracotomy (p=0.490). VATS, video-assisted thoracoscopic surgery

Mortality, which occurred within 30 days, represented 3.2 of the cases. Mortality within 30 days after surgery was found in two patients: one among those who underwent VATS and one among those who underwent thoracotomy. One died from bacterial pneumonia in the VATS group, and one died from sepsis in the thoracotomy group. The mortality rate was 0.9% in VATS patients compared to 2.3% in thoracotomy patients. However, this finding did not show statistical significance (p=0.490). See the comparison shown in Figure 1.

**Figure 2:**
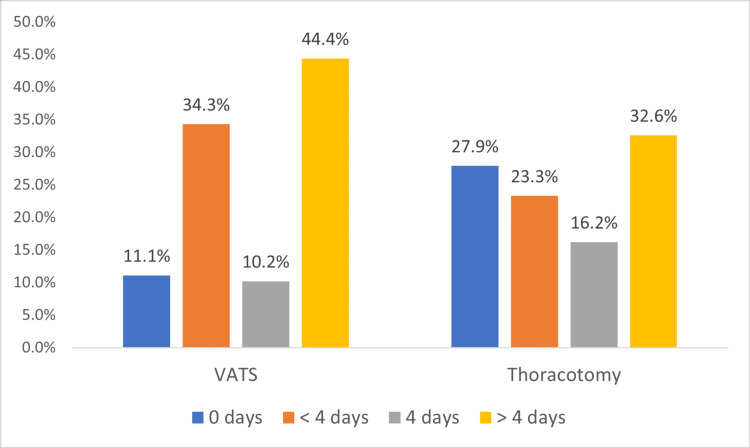
Comparison between the duration of postoperative opioid use for each type of surgery (p=0.033).

Regarding duration of opioid medications (tramadol, morphine, and pethidine), the proportions of patients who needed opioid medications for more than four days were higher in the VATS group than in the thoracotomy group (44.4% vs. 32.6%). Furthermore, those who needed opioid medications for less than four days were higher in the VATS group than in the thoracotomy group (34.3% vs. 23.3%). The association between surgery type and opioid medications was statistically significant (p=0.033). See the results in Figure 2.

## Discussion

PPCs are prevalent following thoracic surgery and can result in significant morbidity and mortality. In recent decades, the advent of minimally invasive surgery has brought about a paradigm shift in several surgical disciplines. Among these, VATS lobectomy has emerged as a transformative technique since its initial description in the early 1990s [11]. Multiple investigations have demonstrated superior perioperative outcomes for VATS lobectomy relative to conventional thoracotomy including reduced incidence of arrhythmias, pneumonia, pain, and lower levels of inflammatory biomarkers [12,13].

The objective of this study was to assess and compare the occurrence of PPCs in patients who underwent thoracotomy and thoracoscopic surgeries. The results of the study demonstrated that patients who underwent thoracoscopic surgeries had a lower incidence of PPCs within the 30-day period after surgery compared to those who underwent thoracotomy (19.4% vs. 23.3%). However, statistical analysis did not establish a significant difference between the two groups (RR=1.19, p=0.657). These findings are consistent with earlier research that reported a reduced occurrence of PPCs in patients who underwent thoracoscopic surgery compared to thoracotomy [14-16].

Al-Ameri et al. reported that patients who underwent open thoracotomy had a higher incidence of transfusions (5.0% vs. 1.4%, p=0.008) and pneumonia (5.5% vs. 0.6%, p=0.002) compared to those who underwent VATS [14]. Furthermore, a review of 34 studies showed a reduced risk of pulmonary complications (risk ratio = 0.70) with thoracoscopic surgery compared to thoracotomy [17]. The lower incidence of PPCs in thoracoscopic surgery can be attributed to several factors. One potential explanation is that thoracoscopic surgery leads to less trauma to the surrounding tissue and a smaller incision, which results in decreased postoperative pain and a quicker return to normal respiratory function [18]. Additionally, thoracoscopic surgery allows for improved visualization of the surgical field and greater precision during the procedure, which may result in a lower incidence of lung injury and subsequent PPCs [19]. However, these findings have been contradicted in several other studies. The study by Gopaldas et al. investigated postsurgical outcomes in 13,619 patients who underwent VATS and thoracotomy [20]. Their findings showed that pulmonary complications were comparable between VATS and thoracotomy groups (32.2% vs. 31.2%; p=0.55). Similarly, in a nationwide propensity-matched cohort study involving 13,027 patients, it was found that VATS did not demonstrate a significant reduction in the risk of unplanned readmission compared to open thoracotomy (20.7% vs. 21.9%, hazard ratio=1.03) [21]. Most of the resources indicated that most frequent complications associated with readmission at 30 days, 90 days, and 12 months were pulmonary complications without difference between VATS and open thoracotomy (10.9% vs 11.1% at 12 months, p=0.2510) [21].

Additionally, the 30-day mortality rates were 2.3% in the open thoracotomy group and 0.9% in the VATS group (p=0.490), while the 90-day mortality rates were 1.7% in the open thoracotomy group compared to 0.3% in the VATS group (p-value=0.09) [14]. These results were comparable to the present study as mortality was higher in the thoracotomy group compared to VATS (18.6% vs. 12%).

The length of hospitalization is a crucial indicator of a hospital’s management effectiveness. Prolonged hospital stay negatively impacts the healthcare system, raises the risk of hospital-acquired infections, increases hospital expenses, worsens psychological well-being, and results in bed shortages. Besides, when comparing the length of hospital stays following VATS and thoracotomy, the majority of patients (34.9%, p=0.013) who underwent thoracotomy spent more than 10 days. On the contrary, most patients (32.4%, p=0.013) who underwent VATS stayed for three days or less. Yang et al. showed similar outcomes, which demonstrated shorter hospital lengths of stay in the VATS group (five vs. six days, p<0.001) [22]. Another recent study, published in 2022, proved that hospital stay was longer in the thoracotomy group than in the VATS group (five to seven days vs. four to seven days, p<0.001) [23]; this fact makes VATS preferable over the thoracotomy.

ICU stays that are too long are linked to greater mortality rates, longer hospital stays, and worse long-term survival rates [24]. Based on the literature [13,25], thoracotomy is expected to have more ICU admissions and longer stay. A study conducted in Poland published in 2013 compared the ICU admission between thoracotomy and VATS and found that following thoracotomy, ICU admissions were nearly two times more common than following VATS [26]. Correspondingly, this study showed that patients who underwent thoracotomy had twice as much ICU admission (65.1%, p<0.001) compared to the VATS group (33.3%). In the VATS group, the intensive care unit admission for seven days or less was 72.2%, while in the open thoracotomy group, it was less (69.9%). This was also demonstrated in another study, as there was no difference in the length of ICU stay in both groups [13].

Moreover, endotracheal intubation after the surgical procedure is one of the factors that can affect the outcomes of procedures. In this study, a minority (6.50 %, P<0.001) of the patients who underwent VATS had intubation, while in the thoracotomy group, 32.60% (p<0.001 ) were intubated. Furthermore, the length of intubation is another aspect that can reflect on the outcomes. A study by Merritt et al. from the USA between 2013 and 2017 demonstrated that VATS is associated with less prolonged intubation when compared to thoracotomy (0.6% vs. 2.7%, p=0.001) [25]. However, in this study, the length of intubation between VATS and thoracotomy proved the opposite, but it was not statistically significant (p-value=0.687). Data indicate that postoperative intubation for 24 hours or less in patients who underwent VATS surgery vs open thoracotomy were 14.3 %and 21.4% respectively. While those who were intubated for more than 24 hours were 85.7% and 71.4%, respectively. These differences in percentage may be attributed to the difference in sample size in the two surgical groups.

After thoracic surgery, a chest tube is frequently inserted to eliminate fluid during healing, and, although insignificant, most of the patients in both groups (VATS and thoracotomy) had chest tubes inserted (95.40% vs. 95.30%, respectively). Comparing the length of chest tube stay in both groups, most of the patients in the VATS group had a chest tube for three to seven days (62.1%, =0.006), and the same goes for the thoracotomy group (70.7%, p=0.006). In contrast, there was a significant difference between both groups regarding chest tube duration according to a study published in October 2022. It showed that chest tube insertion duration was longer in the thoracotomy group than the VATS group [23]. The same results were reported in a 2020 study, which found that there was an early chest tube removal in the VATS group compared to the thoracotomy group [27].

Effective pain management following thoracic surgery remains a significant challenge. Thoracotomy is often associated with significant postoperative pain and discomfort; therefore, VATS was developed as a less invasive alternative [28]. However, even VATS is associated with significant pain. Systemic opioid analgesics are frequently used to manage postoperative pain and upon hospital discharge. In the present study, there was a statistically significant association between surgery type and opioid duration (P-value=.033). We found that patients who needed opioid medications for more than four days were higher in the VATS group than in the thoracotomy group (44.4% vs. 32.6%). In our study, the VATS group required longer time of opioid pain medications of four days or less (34.3%) in comparison to 23.3% in open thoracotomy patients who were given epidural analgesia in most of the cases. However, some studies have reported higher use of opioid medications after surgical procedures in patients who underwent thoracotomy compared to VATS [29,30]. Razi et al. demonstrated that the median use of opioids was significantly lower in patients who underwent thoracoscopy compared to those who underwent thoracotomy (p=0.009 and p=0.27, respectively) [30].

It is worth noting that while thoracoscopy has been shown to be advantageous in reducing the incidence of PPCs, it is not always a suitable approach for every patient or surgical indication. For example, in cases where the tumor is too large or too close to vital structures, or where lymph node dissection is required, a thoracotomy may still be the preferred approach [10].

It is important to note that our study has some limitations, which include its retrospective design, the lack of randomization, and the inclusion of patients from a single center. Future prospective studies with larger sample sizes and multi-center designs are needed to confirm our findings.

## Conclusions

In summary, this study aimed to compare the incidence of PPCs and mortality rates between patients who underwent thoracotomy and thoracoscopic surgeries. Our results revealed that while VATS was associated with a lower incidence of PPCs compared to thoracotomy, the prevalence of such complications did not exhibit a statistically significant difference between the two groups. Furthermore, compared to VATS, the mortality rate within 30 days was twice as high in the thoracotomy group, but no significant variation was noted. Moreover, regarding the postoperative opioid medication, unexpectedly, the need for opioids postoperatively in the thoracotomy group was significantly lower than the VATS group. While prior studies have demonstrated the superiority of VATS over thoracotomy, we cannot definitively assert such superiority in this study. Nonetheless, the selection between thoracoscopic surgery and thoracotomy should be tailored based on several factors including individual characteristics, tumor characteristics, and the surgeon's level of experience and expertise.

This study offers valuable insights into the outcomes of elective thoracotomy and VATS surgeries in the Saudi Arabian population. Future research with larger sample sizes, prospective designs, and multi-center settings should be conducted to validate these findings and investigate potential factors that may impact the outcomes of these surgical modalities.
